# Trajectories of college students' general self-efficacy, the related predictors, and depression: A piecewise growth mixture modeling approach

**DOI:** 10.1016/j.heliyon.2023.e15750

**Published:** 2023-04-25

**Authors:** Xinqiao Liu, Xinyu Ji, Yifan Zhang

**Affiliations:** School of Education, Tianjin University, Tianjin, 300350, China

**Keywords:** General self-efficacy, Depression, Developmental trajectories, College students, Piecewise growth mixture model

## Abstract

General self-efficacy plays a critical role in the development of college students, and mastering the development of students' general self-efficacy is helpful to explain students' behavior and psychological performance. Based on the data from the same cohort of college students for four consecutive years, this study used the piecewise growth mixture model to identify the developmental trajectories of general self-efficacy, built a multinomial logistic regression model to analyze the related predictors on different trajectories, and further compared the differences in depression symptoms in general self-efficacy trajectories. Three trajectories of college students' general self-efficacy were identified: stable-rising (8.7%), stable-decreasing (2.4%), and moderate and stable (88.9%). With the moderate and stable class as the reference, gender and extraversion are the predictors of students in the stable-increasing class; gender, extraversion, mother’s education level, and university tier significantly predict students who fall into the stable-decreasing class. With the stable-increasing class as the reference, gender also has a significant predictive effect on students who belong to the stable-decreasing class. However, age, ethnicity, siblings, hometown location, father’s education level, BMI, sleep, and major were not related predictors. Furthermore, mean differences in depression between latent classes of general self-efficacy trajectories were significant, and the depression scores of the stable-decreasing class were beyond the normal range in the third and fourth years. To promote college students' mental health, we suggest that colleges provide more specific psychological interventions to students based on the classification.

## 1 Introduction

American psychologist Bandura [1] first proposed the concept of self-efficacy and defined it as a subjective judgment of an individual’s ability to complete a behavioral activity before it is carried out. He believed that individuals with high self-efficacy invested more and had a higher probability of success. Wood and Bandura [2] believed that self-efficacy was domain specific, which means that individuals have different levels and types of self-efficacy in different fields. In subsequent research, Bandura (1988) found that self-efficacy in one domain could generalize to other domains [3]. Eden [4] proposed General Self Efficacy and defined it as a person's self-assessment of the overall ability to successfully complete a task under various challenges, which is a relatively stable and generalized belief in one’s capability [5]. As a key variable in educational and psychological studies, general self-efficacy helps explain individuals' differences in motivation, attitude, learning, and task performance [6]. As the last stage of adolescence (10–24 years old), university indicates that individuals' social behaviors and psychological qualities are becoming mature [7]. The trajectories and influencing factors of capability belief perceived during college years are worthy of attention. Additionally, college students have a high incidence of depression, and their general self-efficacy is closely correlated with depression, anxiety and other mental diseases. To cope with this threatening reality, this study will identify the development trajectories and related predictors of the trajectory classes, so as to provide predictive means for college students' mental health education and depression intervention.

Previous studies on general self-efficacy mainly used cross-sectional data [[8], [9], [10]], a few longitudinal studies had insufficient sample sizes [11], and most participants were from the same university [12]. Furthermore, studies noticed the average trajectory of general self-efficacy but ignored the heterogeneity among different groups [13]. Individual conditions [14], family environment [15], perceived social support [16] and other factors are correlated with the differences in general self-efficacy. Empirical studies have shown that individuals' behaviors and psychological traits also have different development trajectories within the group [[17], [18], [19]]. Therefore, it is crucial to identify the developmental trajectories of general self-efficacy among different subgroups of students, which is helpful to provide timely and specific psychological intervention for different groups. Although the trajectories of general self-efficacy have been less well studied, some other relevant evidence has been explored. For instance, Peura et al. [20] examined the trajectories of students' reading self-efficacy and identified four trajectories, including high increasing, average stable, low increasing, and low decreasing. Nightingale et al. [21] conducted follow-up surveys on the emotional self-efficacy of freshmen in Britain, and surveys were conducted at time 1 (three weeks after their initial entry into university), time 2 (3 months after their entry into year 1), and time 3 (6 months after their entry into year 1). Four trajectories were classified, including (1) low, stable adjustment, (2) medium, stable adjustment, (3) high, stable adjustment, and (4) low, increasing adjustment.

The different developmental trajectories of college students' general self-efficacy are caused by a variety of factors, and existing studies have provided some evidence of the correlation between general self-efficacy and other variables, which can be divided into personal factors and environmental factors. Personal factors included gender, age, ethnicity, BMI, sleep, and extroversion. Choi [22] studied the differences in the general self-efficacy of people with different gender roles. The study divided gender roles into four categories, androgynous, masculinity, femininity, and undifferentiated, and discovered that groups with masculine traits reported higher levels of general self-efficacy. Skaret et al. [23] measured the general self-efficacy among 20-year-olds in Norway and stated that women’s self-efficacy was lower than men’s. van Zyl and Dhurup [24] used the Kruskal‒Wallis test to determine the influence of gender, age, and race on self-efficacy, and the results showed that gender, age, and race had no significant effect on self-efficacy. Sekuła et al. [25] evaluated the general self-efficacy of patients with morbid obesity and pointed out that their scores of general self-efficacy had a normal distribution and that the average sten score was 7, which is at a high level. Ovaskainen et al. [26] conducted a cross-sectional study of adults from six regions in Finland and noticed that weaker health-related self-efficacy (HSE) was associated with higher BMI. Benyamini et al. [27] evaluated the differences in self-efficacy before and after weight loss. Although the participants' BMI decreased within 10 weeks, self-efficacy showed no significant difference. Wang et al. [28] found that higher general self-efficacy was associated with sleep quality in a survey of junior college students. In studies of extroversion, Ebstrup et al. [29] identified that extraversion and general self-efficacy had a significant positive correlation.

Environmental factors include siblings, father’s education level, mother’s education level, hometown location, university tier, major, etc. A questionnaire survey of first-year junior middle school students noticed that siblings had no significant impact on general self-efficacy [30], and another study among Chinese college students majoring in nursing reached the same conclusion [31]. A recent study pointed out that parents' self-efficacy significantly predicted adolescents' general self-efficacy, and family socioeconomic status played a positive moderating role [32]. Alfaro et al. [33] surveyed Mexican female college students, and the results indicated that academic support from mothers was positively correlated with self-efficacy and that communication was positively correlated with academic support, while the effects of fathers and siblings were not significant. Isaac et al. [34] explored the self-efficacy of Australian medical students, and the two-factor analysis revealed that women and students from rural areas were associated with higher levels of self-efficacy. In the context of the COVID-19 pandemic, a study in Saudi Arabia discovered that the general self-efficacy of college students during the COVID-19 pandemic was significantly correlated with gender, university tier, and family income [35]. A longitudinal study of college students in China discussed the heterogeneity among subgroups and concluded that the general self-efficacy of students at elite universities increased from freshman to junior year [19]. In the test-oriented context of China, students at nonelite universities were more likely to maintain a lower level of self-efficacy. In terms of professional training, Feng et al. [36] identified that teachers' self-efficacy was significantly improved after completing reading content courses in teacher training. Polizzi et al. [37] investigated the relationship between teacher identity and self-efficacy among 165 teachers from 5 American universities, and the results indicated that teachers in mathematics reported low self-efficacy scores. Overall, the study of humanities probably helps students achieve higher self-efficacy, while the study of more difficult science and engineering is more likely to undermine students' confidence.

The general self-efficacy of college students also has some consequences. Some studies have revealed that general self-efficacy is significantly positively associated with optimism, self-regulation, self-esteem, and other variables but significantly negatively associated with depression and anxiety [8]. Considering the high incidence and harm of depression to college students' physical and mental health, academic development, and interpersonal relationships [[38], [39], [40]], the association between general self-efficacy and depression requires attention. Studies have indicated that differences in students' general self-efficacy are significantly associated with different levels of depression. Mystakidou et al. [41] explored the relationship between general self-efficacy and depression among cancer patients and noticed that higher general self-efficacy was associated with lower levels of depression. Cong et al. [42] also emphasized the predictive effect of self-efficacy on depressive symptoms. Therefore, this study classified the students according to general self-efficacy and detected the group with a high incidence of depression, which has important value for the prediction and prevention of depression among college students.

Considering that school systems and cultures are closely correlated with students' general self-efficacy [43], although much research has been conducted on the predictors of general self-efficacy, longitudinal studies and studies of Chinese samples are still lacking. On this basis, this study used the data set of a longitudinal survey for Chinese college students and used the piecewise growth mixture model, multinomial logistic regression model, and chi-square test to determine the developmental trajectories of college students' general self-efficacy and the predictors for different trajectories, as well as to evaluate the differences in depression symptoms among different trajectories. This study proposes the following three hypotheses.

Hypothesis 1 College students have different trajectories of general self-efficacy.

Hypothesis 2 Gender, age, ethnicity, extroversion, BMI, sleep, siblings, parents' education level, hometown location, university tier, and major are significantly associated with different trajectories.

Hypothesis 2.1 Gender, extroversion personality, sleep, mother’s education level, and university tier are positively correlated with the trajectories.

Hypothesis 2.2 Hometown location, BMI, and major are negatively correlated with the trajectories.

Hypothesis 2.3 Age, ethnicity, siblings, and father’s education level are not correlated with the trajectories.

Hypothesis 3 Mean differences in depression between latent classes of general self-efficacy trajectories are significant.

## 2 Methods

### 2.1 Participants

The data in this study came from the data set of the Beijing College Student Panel Survey (BCSPS), which has been widely used and verified by many studies [19,[44], [45], [46], [47], [48], [49]]. The survey used three-stage Probability Proportionate to Size (PPS) sampling, and the selection was as follows: In the first stage, “university” was determined as the sampling unit, and the schools were divided into 6 groups according to the administrative affiliation and ranking, and then 15 universities were selected from them. In the second stage, “major” was selected as the sampling unit, and 25 majors were randomly selected from three well-known Chinese universities (Tsinghua University, Peking University and Renmin University of China), and 15 majors were randomly selected from other universities. In the last stage, 20 students from each major were randomly selected. The Beijing College Student Panel Survey (BCSPS) tracked two groups of students enrolled in 2006 and 2008 for five consecutive years, collecting data on their socio-demographic characteristics, psychological status, and academic performance once in each academic year. The follow-up rate of this panel data was more than 90% [44]. The first three rounds of the survey were conducted on-site, and the final round was organized online, inviting students to log in to the questionnaire website via a specific code assigned by text message and email [45]. The sample included in this study was 2473 college students enrolled in 2008, including 1166 females and 1307 males.

### 2.2 Measures

#### 2.2.1 General self-efficiency

General self-efficacy was measured by the General Self-Efficacy Scale (GSES) [50]. The scale consists of 10 items, and participants self-report the items in the range of 1 (not at all true) to 4 (exactly true). The total score for the GSES is calculated by summating all the items and ranges from 10 to 40, with higher scores indicating higher general self-efficacy. The Cronbach’s alpha on the GSES from freshman to senior year was 0.865, 0.8682, 0.8999, and 0.9094.

#### 2.2.2 Depression

Depression was measured using the Depression Anxiety and Stress Scale-42 (DASS-42) [51], which contains 14 questions. The Depression scale assesses dysphoria, hopelessness, devaluation of life, self-deprecation, lack of interest/involvement, anhedonia, and inertia. Participants self-report each item on a scale of 0 (which did not apply to me at all) to 3 (which applied to me very well, or most of the time). The total score is calculated by adding all the items together, and higher scores indicate more severe symptoms. On the depression scale, “normal” corresponds to a score of 0–9, “mild” to a score of 10–13, “moderate” to a score of 14–20, “severe” to a score of 21–27, and “extremely severe” to a score of 28+. Cronbach's alpha for depression in the four years was 0.8913, 0.8959, 0.9281, and 0.9436.

#### 2.2.3 Related predictors

Related variables were collected in the baseline survey, including age (continuous variable, age at the first collection), gender (dichotomous variable, female = 0, male = 1), ethnicity (dichotomous variable, Han = 0, minority = 1), extraversion (continuous variable), siblings (dichotomous variable, yes = 1, no = 2), BMI (categorical variable, normal = 1, low = 0, high = 2, normal as the reference group), sleep (categorical variable, normal = 1, low = 0, high = 2, normal as the reference group), hometown location (dichotomous variable, urban = 1, rural = 0), university tier (dichotomous variable, “211” universities = 1, other universities = 0), and major (dichotomous variable, engineering/science/agriculture = 1, social sciences/humanities = 0).

### 2.3 Data analysis

First, Mplus 8.3 was used in this study for the analysis of the piecewise growth mixture model (PGMM) [52,53]. Since sophomore year is widely considered to be a transition in higher education, Freedman coined the term “sophomore slump” to describe the period in which sophomores were prone to be confused and depressive [54]. Therefore, we set the sophomore year as the turning point of the PGMM. Combined with the theoretical research of general self-efficacy, Akaike Information Criteria (AIC), Bayesian Information Criteria (BIC), Sample Size Adjusted BIC (SABIC), Lo-Mendel-Rubin Likelihood Ratio Test (LMR-LRT), Bootstrapped Likelihood Ratio Test (B-LRT) and entropy were used to test the fit of the models and determine the model that best explained the trajectories of general self-efficacy. Smaller AIC, BIC, and SABIC values indicated a better model fit [55,56]. LMR-LRT and BLRT were used to compare two adjacent models, and significant p values (<0.05) indicated that the k model fit better than the k-1 class model [57]. Entropy ranged from 0 to 1, with values closer to 1 indicating more accurate classification [58].

Second, this study used Stata 16.0 for multinomial logistic regression analysis to explore the related predictors of the different trajectories of general self-efficacy.

Finally, the Kruskal‒Wallis test was used to compare whether depression had significant differences among trajectories.

## 3 Results

### 3.1 Determination of trajectory classes of general self-efficacy

Based on six fitting indices and the existing research, we determined the trajectory classes of college students' general self-efficacy (Table 1). The AIC, BIC, and SABIC results indicated that the 3-class model fit best. The B-LRT likelihood ratio of the 3-class model was significant when α = 5% and the entropy was 0.82. Considering the sample size ratio of each class, the 3-class model was the most suitable in terms of theoretical interpretability.

**Table 1:** Fit indices of trajectories with 2–4 latent classes.

	2 class model	3 class model	4 class model
AIC	53,321	53,256	53,232
BIC	53,420	53,378	53,377
SABIC	53,366	53,311	53,298
LMR-LRT	*p* < 0.001	p = 0.069	*p* = 0.546
B-LRT	*p* < 0.001	*p* < 0.001	*p* < 0.001
Entropy	0.97	0.82	0.85
Mixture proportion (%)	98.7	88.9	89.2
	1.3	8.7	8.1
		2.4	2.1
			0.6

*Note:* AIC = Akaike Information Criterion; BIC = Bayesian Information Criterion; SABIC = Sample Size Adjusted BIC; LMR-LRT = Lo Mendel-Rubin Adjusted Likelihood Ratio Test; B-LRT = Bootstrapped Likelihood Ratio Test.

### 3.2 Trajectory classes of general self-efficacy

**Fig. 1:**
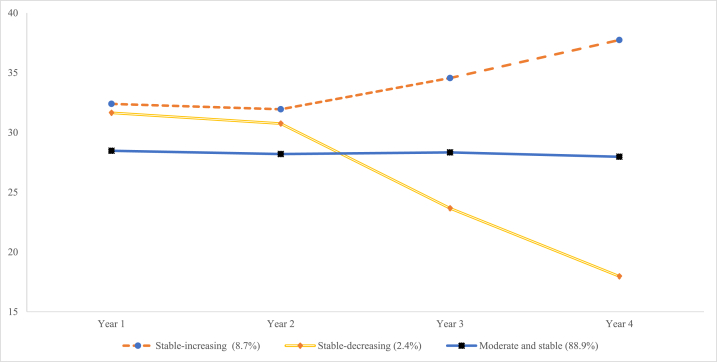
Piecewise growth mixture model trajectory of general self-efficacy.

Fig. 1 shows the trajectories of general self-efficacy. The first class, accounting for 8.7% of the students, remained stable from the freshman year (32.394) to the sophomore year (31.942) and then increased from the junior year (34.556) to the senior year (37.738). This class was named “steady-increasing”. The second class accounted for 2.4%, and the trajectory was stable from the freshman year (31.644) to the sophomore year (30.745), followed by a decline from the junior year (23.655) to the senior year (17.962), and it was termed the “stable-decreasing”. The third class, accounting for 88.9%, was characterized by stable and moderate levels of general self-efficacy over the four years from freshman year (28.468), sophomore year (28.193), junior year (28.332) to senior year (27.967), and we named it “moderate and stable”.

The slope and intercept of each model are shown in Table 2. In the stable-increasing class, the general self-efficacy decreased from the freshman year to the sophomore year, with the decrease not significant (S1 = −0.388, p = 0.449), but the general self-efficacy increased significantly from the sophomore to the senior year (S2 = 2.436, p < 0.001). In the stable-decreasing class, the general self-efficacy showed a slightly decreasing trend from the freshman year to the sophomore year, and this trend was not significant (S1 = −0.766, p = 0.520), while the decrease was significant from sophomore to senior year (S2 = −5.787, p < 0.001). In the moderate and stable class, the overall developmental trajectory was relatively stable. The general self-efficacy decreased slightly from freshman to sophomore year, with the decrease not significant (S1 = −0.179, p = 0.086), and the decrease was also not significant from sophomore to senior year (S2 = −0.086, p = 0.163).

**Table 2:** Intercept and slope of latent classes.

	Stable-increasing (n = 216)			Stable-decreasing (n = 59)			Moderate and stable (n = 2197)		
	Means	SE	*p* value	Means	SE	*p* value	Means	SE	*p* value
I	33.706	0.341	0.000	24.544	0.895	0.000	28.180	0.089	0.000
S1	−0.388	0.513	0.449	−0.766	1.191	0.520	−0.179	0.104	0.086
S2	2.436	0.253	0.000	−5.787	1.221	0.000	−0.086	0.062	0.163

*Note:* I = intercept; S1 = slope of the first phase during year 1 to year 2; S2 = slope of the second phase during year 2 to year 4; SE = standard error.

Hypothesis 1 was verified and identified three developmental trajectories of college students' general self-efficacy.

### 3.3 Related predictors of trajectory classes

The study used a multinomial logistic regression model to identify the related predictors for different trajectory classes of general self-efficacy (Table 3). Taking the moderate and stable class as the reference, the comparison of relative risk rates and p values with the stable-increasing class indicated that men (RRR = 4.39, p = 0.003) and extrovertion (RRR = 1.31, p = 0.021) had statistically significant impacts. In other words, men and extroverted students were more likely to belong to the stable-increasing class. Increasing each rank of extroversion improved the likelihood of belonging to the stable-increasing class by 31%.

**Table 3:** Multinomial logistic regression analyses of predictors for classes of general self-efficacy trajectories.

	Stable-increasing (vs Moderate and stable)			Stable-decreasing (vs Moderate and stable)			Stable-decreasing (vs Stable-increasing)		
	RRR	*95% CIs*	*p* value	RRR	*95% CIs*	*p* value	RRR	*95% CIs*	*p* value
Gender: Men (vs Women)	4.39	1.63–11.82	0.003	1.39	1.13–1.73	0.002	3.15	1.16–8.54	0.024
Age	1.22	0.83–1.78	0.304	1.01	0.90–1.12	0.896	1.21	0.83–1.78	0.328
Ethnicity: Minority (vs Han)	0.56	0.13–2.39	0.433	1.06	0.79–1.42	0.687	0.53	0.12–2.27	0.390
Siblings: No (vs Yes)	0.86	0.35–2.10	0.734	1.09	0.85–1.38	0.499	0.79	0.32–1.95	0.606
Extroversion personality	1.31	1.04–1.65	0.021	1.22	1.15–1.29	0.000	1.08	0.85–1.36	0.528
Hometown location: Urban (vs Rural)	1.90	0.63–5.73	0.254	1.16	0.87–1.54	0.319	1.65	0.54–5.04	0.383
Father's education level	1.10	0.95–1.27	0.222	1.02	0.98–1.06	0.295	1.07	0.92–1.25	0.350
Mother's education level	0.95	0.83–1.07	0.397	1.04	1.00–1.08	0.034	0.91	0.80–1.04	0.153
BMI									
Low (vs Normal)	1.81	0.73–4.48	0.203	0.91	0.71–1.16	0.434	1.99	0.79–5.01	0.142
High (vs Normal)	1.33	0.43–4.06	0.618	1.32	0.96–1.83	0.092	1.00	0.33–3.10	0.995
Sleep									
Low (vs Normal)	2.04	0.75–5.53	0.163	1.09	0.80–1.48	0.573	1.87	0.68–5.14	0.228
High (vs Normal)	1.47	0.43–4.06	0.542	1.08	0.76–1.54	0.672	1.36	0.39–4.79	0.630
Elite university: Yes (vs No)	0.53	0.24–1.14	0.104	0.80	0.65–0.98	0.034	0.66	0.30–1.44	0.299
Major: Science/Engineering (vs Social sciences/Humanities)	2.02	0.85–4.82	0.113	0.89	0.72–1.09	0.257	2.28	0.95–5.47	0.066

*Note:* RRR = relative risk ratio; 95% CIs = 95% confidence intervals.

Comparing the moderate and stable class with the stable-decreasing class, the relative risk ratios and p values for males (RRR = 1.39, p = 0.002), extrovertion (RRR = 1.22, p < 0.001), mother’s education level (RRR = 1.04, p = 0.034), and university tier (RRR = 0.80, p = 0.034) were statistically significant, which implied that men, extroverted students, students with highly educated mothers, and students from nonelite universities were more likely to belong to the stable-decreasing class of general self-efficacy. For each increase in the range of extroversion, the likelihood of falling into the stable-decreasing class increased by 22%. Each improvement in the mother’s education level increased the likelihood of belonging to the stable-decreasing trajectory of general self-efficacy by 4%. Compared with students from nonelite universities, the likelihood of students from elite universities belonging to the stable-decreasing class decreased by 20%.

The study also compared the stable-increasing class with the stable-decreasing class and discovered that the relative risk ratios and p value for men (RRR = 3.15, p = 0.024) were statistically significant, suggesting that men were more likely to be classified as the stable-decreasing class. Age, ethnicity, siblings, extroversion, hometown location, father’s education level, mother’s education level, BMI, sleep, university tier, and major had no significant effect.

The above verified Hypothesis 2 that an individual’s gender, extroversion, mother’s education level, and university tier are the related predictors of the general self-efficacy trajectories among college students.

### 3.4 Mean differences in depression between latent classes of general self-efficacy trajectories

Mean differences in depression between latent classes of general self-efficacy trajectories are shown in Table 4, with significant differences in depression scores at different years. In the first two rounds of investigation, the moderate and stable classes had higher depression scores. In the latter two rounds of investigation, the depression score of the stable-decreasing class was higher, while the depression scores remained low when the students' general self-efficacy was stably increasing.

**Table 4:** Mean differences in the symptoms of depression between latent classes of general self-efficacy trajectories.

	Stable-increasing^a^		Stable-decreasing^b^		Moderate and stable^c^		
	Means	SD	Means	SD	Means	SD	
Depression at year 1	5.764	6.307	6.763	6.624	7.585	6.508	a < b < c
Depression at year 2	6.083	6.210	7.873	6.532	7.948	6.706	a < b < c
Depression at year 3	5.179	6.451	11.224	9.181	8.050	7.118	a < c < b
Depression at year 4	4.296	6.799	11.472	9.003	7.524	7.067	a < c < b

*Note:* a = stable-increasing; b = stable-decreasing; c = moderate and stable; SD = standard deviation.

Classes had significant differences in depression scores at year 1 (χ2 = 27.742, p < 0.001), and the moderate and stable class had the highest average score (Mean = 7.585, SD = 6.508), which was significantly higher than the stable-decreasing class (Mean = 6.763, SD = 6.624) and the stable-increasing class (Mean = 5.764, SD = 6.307). At year 2, mean differences in depression scores were also significant among the classes (χ2 = 21.104, p < 0.001), and the moderate and stable class had a higher average score (Mean = 7.948, SD = 6.706) than the stable-decreasing class (Mean = 7.873, SD = 6.532) and the stable-increasing class (Mean = 6.083, SD = 6.210). At year 3, the depression scores were still significantly different among the classes (χ2 = 57.039, p < 0.001). The stable-decreasing class (Mean = 11.224, SD = 9.181) obtained the highest average score, and it was significantly higher than that of the moderate and stable class (Mean = 8.050, SD = 7.118) and the stable-increasing class (Mean = 5.179, SD = 6.451). At year 4, the depression scores remained significantly different among the classes (χ2 = 76.882, p < 0.001). Students in the stable-decreasing class (Mean = 11.472, SD = 9.003) experienced more severe depression symptoms compared with the moderate and stable class (Mean = 7.524, SD = 7.067) and the stable-increasing class (Mean = 4.296, SD = 6.799). Overall, students in the stable-increasing class and the moderate and stable class remained in the normal (0–9) state, but students in the stable-decreasing class suffered from mild depression in the third and fourth years.

Hypothesis 3 was verified, and the mean differences in depression between latent classes of general self-efficacy trajectories were significant.

## 4 Discussion

This study used data from a longitudinal survey over four consecutive years to analyze the developmental trajectories of general self-efficacy among Chinese college students, explore the related predictors of different trajectories, and evaluate the differences in depression symptoms among different trajectories. The results provide empirical evidence for the trajectories of general self-efficacy and some inspiration to predict and prevent depression symptoms among adolescents.

First, the results of the PGMM supported Hypothesis 1. Based on fit indices such as AIC, BIC, SABIC, LMR-LRT, B-LRT, and entropy and relevant theoretical research on general self-efficacy, the developmental trajectories of general self-efficacy were divided into three classes. In the stable-increasing class, the general self-efficacy maintained a stable and moderate level during year 1 - year 2 and increased during year 2 - year 4. In the stable-decreasing class, students' general self-efficacy was stable and moderate during year 1 – year 2 but decreased during year 2 - year 4. In the moderate and stable class, the general self-efficacy remained at a moderate and stable level from year 1 - year 4. Students in different classes held a similar level of general self-efficacy in the first two years, but differences appeared in the sophomore year and led to different developmental directions. Therefore, sophomore year deserves more attention. Since the PGMM of general self-efficacy had not been studied before, we compared other relevant findings. The trajectories of reading self-efficacy were divided into four classes: high increasing, average stable, low increasing, and low decreasing [20]. Our results were similar to this classification, but they did not consider the heterogeneity at different stages and only classified the average trajectories. Another longitudinal study on the emotional self-efficacy of British students in the first year of college divided into four trajectories, and three of them eventually turned to stable adjustment, which was similar to our finding of self-efficacy in the freshman year [21]. In general, to complement the existing studies, this study proved that the developmental trajectories of college students were different and further identified three trajectories.

Second, the related predictors of different trajectories of general self-efficacy were analyzed from the dimensions of gender, extroversion, mother’s education level, and university tier, verifying Hypothesis 2. Specifically, men and extroverted students were more likely to belong to the stable-increasing class than to the moderate and stable class. Men, students with highly educated mothers, and students from nonelite universities were more likely to fall into the stable-decreasing class than to the moderate and stable class. Men’s general self-efficacy was more likely to be stable and decrease compared with being stable and increasing. Many previous studies have pointed out that men’s self-efficacy is higher than that of women [22,23], which is consistent with the findings in this study that men’s self-efficacy is more likely to be stable and increase. Inconsistent with other studies, van Zyl and Dhurup [24] stated that the gender differences in general self-efficacy were not significant. This contradiction might be caused by the different sources and regions of the research objects. In this study, men’s general self-efficacy was more likely to increase than women’s, which was probably related to the gender differences in labor division in the Chinese context. Men were also more likely to be in the stable-decreasing class, but the unstable performance of men’s general self-efficacy has not been discovered in previous studies. The possible reason is that men’s general self-efficacy more comes from external motivation, while women’s general self-efficacy has a higher mean value in internal motivation [59]. Perceived overall self-efficacy and motivation to learn in high school teenagers.). External motivation depends on the external environment, which is unstable. When male college students receive external rewards in academic or other aspects (such as excellent grades, scholarships, etc.), higher external motivation prompts higher self-efficacy. In contrast, when they experience failure, this “punishment” will decrease their self-efficacy [59]. Furthermore, the results suggested that extroverted students were more likely to fall into the stable-increasing class than the moderate and stable class, which was consistent with the findings of Ebstrup et al. [29]. Extroverted people treat stress as a challenge rather than a threat [60], and they generally believe in their ability to overcome difficulties, so extroverted personality has a significantly positive association with general self-efficacy. However, extroverted students are also more likely to belong to the stable-decreasing class than to the moderate and stable class. The explanation for this is that extroverted people are probably more susceptible to being influenced by the external environment, and adverse changes in the environment may lead to the decline of their general self-efficacy.

Students with highly educated mothers and from nonelite universities were more likely to experience general self-efficacy from stable to decreasing. Parental knowledge has been proven positively associated with general self-efficacy in previous studies [32,33], and perceived parental support can provide adolescents with the foundation that enables them to explore their environment and face challenges with more confidence, contributing to improving their self-efficacy. This is not consistent with our findings, and two reasons are listed. First, the research objects come from different regions. In China, fathers and mothers play different roles in families. Mothers often have more direct emotional and life contact with their children, while fathers are mainly responsible for the family economy. Given the differences in the division of parental roles, the conclusions may be different among the countries. Second, higher education changes the labor division. Although both the father and the mother are both providing love and affection for children, mothers were mainly responsible for rearing the children in the previous labor division of Chinese families. In recent years, women have become more educated, and more families have two working parents. Therefore, students who have difficulty getting support from their parents are more likely to fall into the stable-decreasing class of general self-efficacy. Universities in China can be classified into three tiers based on government support and their advantages. The first tier consists of 39 universities under Project 985, the second tier includes 73 universities under Project 211, and the third tier is made up of other universities. We classified universities under Project 211 (including universities under Project 985) as elite universities, which received larger amounts of public funding and support, while students attending nonelite universities received fewer resources, which might lower students' general self-efficacy. This finding is consistent with existing conclusions in China [19]. The study analyzed the influence of personal factors (gender, extroversion) and environmental factors (mother’s education level, university tier) on the different developmental trajectories of general self-efficacy, which was of great significance for predicting and intervening in the developmental trajectories of general self-efficacy.

The findings verified Hypothesis 3 that mean differences in depression between latent classes of general self-efficacy trajectories were significant. The depression scores of the steady-increasing class over the four years were between 4.296 and 5.764, within the normal range. The depression scores of the moderate and stable classes ranged from 7.524 to 8.050, also in the normal range, but were higher than those of the stable-increasing class. In the stable-decreasing class, the depression scores were between 6.763 and 11.472. The depression scores exceeded the normal range in the last two years and were much higher than those in the other two classes. Among the three classes, the depression scores of the stable-increasing class were the lowest over the four years, which was consistent with previous studies showing that general self-efficacy had a negative predictive effect on depression [9,41,42]. In other words, people with higher general self-efficacy are less prone to depression, and people with low self-efficacy have a high incidence of depression. It is worth noting that in the third and fourth years, students in the stable-decreasing class suffered from mild depression, which could be explained by the lack of mental health education in universities and students' less attention to their mental state. This reminds universities to be vigilant and strengthen mental health education, and it is necessary to carry out interventions for juniors and seniors with low general self-efficacy and prevent students from going to extremes.

## 5 Limitations

Some limitations exist in this study. First, the self-report questionnaire was adopted, which may lead to some bias in the results. Second, all the subjects were college students in Beijing, China. Whether the conclusion can be extended to all college students in China remains to be determined with caution. Third, this study measured extroversion through self-report and did not use a specific questionnaire.

## 6 Conclusions

First, this study confirmed that there were three developmental trajectories of general self-efficacy during college years, namely, stable-increasing, stable-decreasing, and moderate and stable. In the stable-increasing class, students' general self-efficacy did not change significantly from the freshman year to the sophomore year but increased significantly from the sophomore year to the senior year. In the stable-decreasing class, students' general self-efficacy also remained stable from the freshman to the sophomore year but significantly decreased from the sophomore to the senior year. In the moderate and stable class, students' general self-efficacy remained moderate and did not change significantly from freshman to senior year.

Second, with the moderate and stable class as the reference, gender and extraversion are the predictors of students in the stable-increasing class, and gender, extraversion, mother’s education level, and university tier significantly predict students who fall into the stable-decreasing class. With the stable-increasing class as the reference, gender also has a significant predictive effect on students who belong to the stable-decreasing class.

Third, this study identified that mean differences in depression between latent classes of general self-efficacy trajectories were significant, and the depression scores of the stable-decreasing class were beyond the normal range in the third and fourth years.

Overall, it is suggested that universities classify students according to their general self-efficacy and carry out specific interventions for different classes. Meanwhile, timely measures should be taken for students with low self-efficacy in the junior and senior years to ensure the mental health of college students.

## Declarations

### Author contribution statement

Xinqiao Liu: Conceived and designed the experiments; Wrote the paper.

Xinyu Ji: Performed the experiments; Analyzed and interpreted the data; Wrote the paper.

Yifan Zhang: Analyzed and interpreted the data; Wrote the paper.

## Data availability statement

Data will be made available on request.

## Declaration of interest’s statement

The authors declare the following conflict of interests: Dr. Xinqiao Liu is an advisory board member of Heliyon's Education section. The other two authors declare no conflicts of interest.

## Funding statement

This study was funded by Tianjin philosophy and social science planning project “Research on Value-added Evaluation of Career Adaptability for Engineering Students Oriented towards Outstanding Engineers”, grant number TJJXQN22-001.
